# Public data and open source tools for multi-assay genomic investigation of disease

**DOI:** 10.1093/bib/bbv080

**Published:** 2015-10-10

**Authors:** Lavanya Kannan, Marcel Ramos, Angela Re, Nehme El-Hachem, Zhaleh Safikhani, Deena M.A. Gendoo, Sean Davis, David Gomez-Cabrero, Robert Castelo, Kasper D. Hansen, Vincent J. Carey, Martin Morgan, Aedín C. Culhane, Benjamin Haibe-Kains, Levi Waldron

**Keywords:** multiple assays (multi-assays), public data, bioconductor, integrative genomics, cancer, pharmacogenomics, omics

## Abstract

Molecular interrogation of a biological sample through DNA sequencing, RNA and microRNA profiling, proteomics and other assays, has the potential to provide a systems level approach to predicting treatment response and disease progression, and to developing precision therapies. Large publicly funded projects have generated extensive and freely available multi-assay data resources; however, bioinformatic and statistical methods for the analysis of such experiments are still nascent. We review multi-assay genomic data resources in the areas of clinical oncology, pharmacogenomics and other perturbation experiments, population genomics and regulatory genomics and other areas, and tools for data acquisition. Finally, we review bioinformatic tools that are explicitly geared toward integrative genomic data visualization and analysis. This review provides starting points for accessing publicly available data and tools to support development of needed integrative methods.

## Introduction

The falling cost of genomic assays has enabled more comprehensive molecular profiling, also referred to as ‘omics', of biological specimens for developing systems approaches to molecular biology, studying disease etiology and improving treatment outcomes [1]. The growth of ‘multi-assay' genomic experiments is led by major public projects such as The Cancer Genome Atlas (TCGA) and the International Cancer Genome Consortium (ICGC) [2], but smaller-scale projects are increasingly being undertaken by individual laboratories and deposited in public databases such as the National Center for Biotechnology Information (NCBI) Gene Expression Omnibus (GEO) [3] and European Bioinformatics Institute (EBI) ArrayExpress [4] databases. We define ‘integrative' analysis in this context as analysis that spans multiple molecular data types, including, for example, somatic mutations, copy number, DNA methylation, messenger RNA (mRNA) expression and protein abundance. It can include other data types such as metabolite abundance and microbiome profiling, as well as metadata such as clinical outcome and tumor pathology in cancer studies. Even though generation of multi-assay molecular data sets has become common, integrative data analysis remains a significant challenge and has been limited primarily to those laboratories with substantial bioinformatic expertise (e.g. [5–7]).

The objective of this article is to accelerate the development of bioinformatic and statistical methodology that facilitate the integrative analysis of multi-assay genomics experiments. We do this by reviewing appropriate data from various fields that are in the public domain and that can be used for the development of new analytical approaches. Sources of these data include large consortial projects in clinical oncology, pharmacogenomics and other cell line perturbation experiments, population genomics and regulatory genomics. We review additional data available from smaller experiments performed by individual laboratories that have deposited data in public databases. We also review tools that simplify acquisition of these data, primarily for TCGA and for GEO. Finally, we conclude by summarizing the current state of tools for integrative genomic data analysis and discussing the gaps left by these tools.

## Clinical oncology

Major data-generating projects provide numerous genomic assays mostly from resected primary tumors, along with clinical and histopathological data. They represent the most comprehensive collections of multi-assay genomic data sets currently available, and important test cases for related methodological development.

### The Cancer Genome Atlas

**Figure 1.**
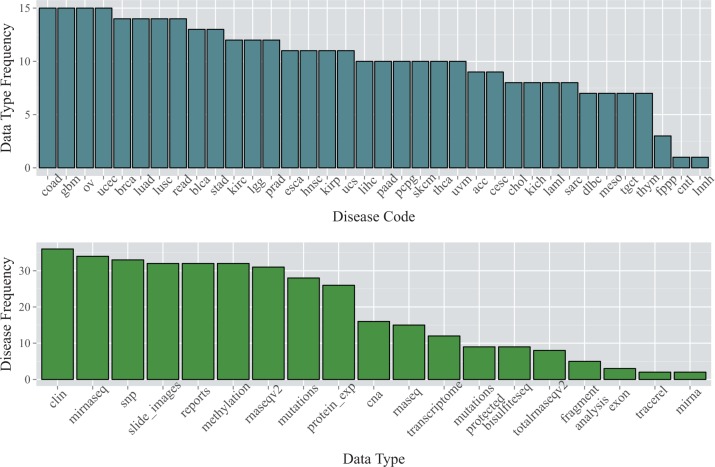
Data and cancer types provided by TCGA. The top barplot shows the number of data types available for each of the 36 cancer types (key provided as Supplementary Table S1) as of January 2015. Cancer types with fewer data types are still in the process of data collection. The lower barplot shows the number of cancer types for which each data type is available (key provided as Supplementary Table S2).

Under the umbrella organization of the National Institutes of Health (NIH), the TCGA project is a joint collaboration between the National Cancer Institute (NCI) and the National Human Genome Research Institute (NHGRI) aimed at understanding the molecular basis of cancer. It is the largest available resource for multi-assay cancer genomics data, and aims to profile over 11 000 patients representing 36 cancer types, using up to 15 genomic assays per tumor, in combination with clinical and pathological annotations. The TCGA PanCanAtlas project has described integrative analysis across 12 tumor types (http://www.nature.com/tcga/). Most cancer types are still in progress; a snapshot at the time of writing of the number of data types currently available per cancer type is provided in Figure 1. Numerous tools, summarized in Table 1, have been developed to simplify the daunting process of data acquisition from this project.

**Table 1.** TCGA data acquisition tools

Name and citation	Description	Download type	Data analysis integration	Data level	Software implementation
RTCGAToolbox [8]	R package for downloading preprocessed data	Bulk	High	3–4	'RTCGAToolbox' - Bioconductor Package
firehose_get [9]	Unix command line tool	Bulk	Low	1–4	Command line, wget
Linked TCGA [10]	5 star-linked open data via SPARQL endpoints	Bulk	Low	3	Resource Description Framework (RDF) and SPARQL query endpoints
MSKCC cBioPortal [11, 12]	R package and Web interface to the MSKCC Cancer Genomics Data Server	Limited	High	3–4	'cgdsr'—R Package
UCSC Cancer Genomics Hub [13]	Restricted access tool to raw data files	Bulk	Low	1	GeneTorrent client (gtdownload)
TCGA Assembler [14]	R script files for downloading preprocessed data	Bulk	Medium	1, 3	Collection of R scripts
Synapse client [15]	Download within R using Synapse syntax (credentials required)	Limited	Medium	1–4	'synapseClient'—R Package
TCGA Data Portal [16]	Bulk, table and HTTP-link-based repository	Variable	Low	1–4	Web site and Web client
TCIA Imaging Archive [17]	Repository for medical images of cancer in DICOM format	Bulk	–	1	Web site download

‘Data levels’ are defined by TCGA, varying from 1 (raw data) to 4 (data analysis resulting from multiple samples, such as regions of common copy number variation).

### International Cancer Genome Consortium

The ICGC currently coordinates 55 research projects, which collectively aim at obtaining a complete catalog of alterations that characterize the genome, transcriptome and epigenome in 50 forms of tumor that mainly contribute to the burden of disease in people throughout the world. The ICGC data portal (https://dcc.icgc.org/) provides tools for visualizing, querying and downloading the data released quarterly by the consortium's member projects. It also provides basic analysis.

### Molecular Taxonomy of Breast Cancer International Consortium

The Molecular Taxonomy of Breast Cancer International Consortium (METABRIC) data set (http://molonc.bccrc.ca/aparicio-lab/research/metabric/) contains clinical traits, expression, copy number variation profiles and single nucleotide polymorphism (SNP) genotypes derived from breast tumors collected from participants of the METABRIC trial [18].

### Pharmacogenomics and other perturbation experiments

**Figure 2.**
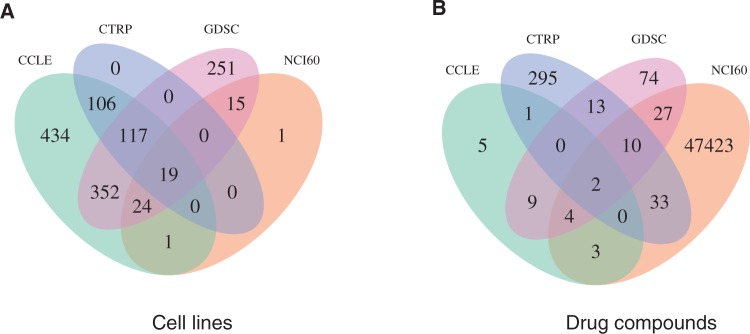
Overlap across publicly available pharmacogenomic data sets. (A) Cell lines that have been molecularly and/or pharmacologically profiled in each study. (**B**) Drug compounds screened in each study. The substantial overlap across large pharmacogenomic studies using different molecular and pharmacological profiling assays enables integrative analysis to define more robust biomarkers of drug response.

Cancer cell lines are widely used as preclinical models to gain mechanistic and therapeutic insight. Common approaches include pharmacogenomics, and application of genetic perturbation reagents (such as shRNAs or cas9/sgRNAs) to silence or knock-out individual genes and identify those genes that affect cell survival. This section describes large-scale molecular and pharmacological characterization of human cancer cell lines for which multi-assay data are publicly available. Substantial overlap exists in both the cell lines and compounds used by these studies, offering still unrealized potential for large-scale integration (Figure 2). Table 2 summarizes these pharmacogenomic and perturbation cell line data sets.

**Table 2.** Multi-assay pharmacogenomic and perturbation cell line data sets

Program name	Number of unique cell lines	Number of unique tissues of origin	Assay	Number of drugs tested	FDA-approved drugs
CMAP	5	4	GE array	1,309	576
L1000	77	15	GE array	20,431	851
NCI60	60	9	GE array, SNP array, RPPI	49,938	201
CGP	727	32	GE array, WXS; SNP array	140	29
CCLE	1036	24	GE array and RNA-seq, WXS/WGS; SNP array	24	8
CTRP	242	17	See CCLE	354	36
CTD2	243	20	DNA-seq, GE array, RPPA, perturbation-based screens, Comparative Genomic Hybridizations	355	35
GDSC	714	14	GE array, genetic mutations	142	
Achilles	216		GE array, genetic mutations, phenotypic information	54,020	
LINCS	356		RNAseq, Proteomics	5,943	

#### The NCI-60 cell line panel

The NCI-60 panel of 59 human tumor cell lines are perhaps some of the most extensively characterized cell lines [19]. Data sets from these cell lines include protein levels, RNA measurements, mutation status, and enzyme activity levels. The molecular and pharmacological data are publicly available on the CellMiner (http://discover.nci.nih.gov/cellminer/) [20] and DTP (http://dtp.cancer.gov/mtargets/mt_index.html) Web sites, respectively. Published studies reported results from integrative analysis of DNA copy number with gene expression levels, and drug sensitivities have been performed [21, 22]. Limited integrative analysis tools are available through CellMiner, for users who wish to investigate the molecular basis of drug response in the NCI-60 cell line panel. The NCI-60 proteomic data can be downloaded from http://wzw.tum.de/proteomics/NCI60/as well as from https://www.proteomicsdb.org.

Numerous projects provide additional pharmacological and perturbation experimental results on the NCI-60 cell lines. Notably, the In Vitro Cell Line Screening Project screens up to 3000 compounds per year for potential anticancer activity on these cell lines. However, this and other experiments using the NCI-60 cell lines are not accessible through CellMiner and require additional curation and processing to enable integrative analysis with the base genomic characterizations. Lack of standardization in cell line and drug names is a challenge that can require careful inspection to resolve [23].

#### The Cancer Cell Line Encyclopedia

The Cancer Cell Line Encyclopedia (CCLE) data set is a compilation of gene expression, copy number and DNA sequencing data from 947 human cancer cell lines. It also comprises the pharmacological profiles for 24 anticancer drugs across 504 of the cell lines (http://www.broadinstitute.org/ccle). Barretina and colleagues demonstrate multi-assay analysis of these samples in identifying genetic, cell-of-origin and gene-expression-based predictors of drug sensitivity using these two data sets [24]. This data set provides a platform to integrate different assays that link pharmacologic vulnerabilities to molecular patterns and to develop new companion tests for targeted chemotherapies [25]. The metadata of pharmacologic profiling and expression microarray describe the inconsistency in names that are used for the cell lines, which may be overcome by writing curation scripts to map cell line names. However, the data set license restricts redistribution of curated versions that would be more readily integrated with other pharmacogenomic data resources.

#### Genomics of Drug Sensitivity in Cancer

Genomics of Drug Sensitivity in Cancer (GDSC) is a dedicated academic research program of the Cancer Genome Project (CGP) to study the therapeutic targets for cancer (http://www.cancerrxgene.org/). The GDSC database (http://www.cancerrxgene.org/) is a public resource containing data from about 75 000 experiments on 142 anticancer drugs across almost 714 cell lines. The compounds studied include cytotoxic chemotherapeutics as well as targeted therapeutics from commercial sources, academic collaborations and from the biotech and pharmaceutical industries. Similar to CCLE, the large collection of cell lines helps to capture genomic heterogeneity underlying human cancer. Drug sensitivity patterns of the cell lines can be correlated with expression data to identify genetic features that are predictive of sensitivity, to identify mutated cancer genes associated with cellular response to available cancer drugs [26].

#### The Cancer Therapeutics Response Portal

Cancer Therapeutics Response Portal (CTRP) provides open access to quantitative sensitivity measurements to a 354-member ‘Informer Set’ of small-molecule probes and drugs, for 242 genetically characterized cancer cell lines. Although CTRP does not provide molecular profiles data, it contains selected cell lines that have been molecularly characterized within CCLE, making it possible to develop new biomarkers of drug response [27]. Although the current implementation of the portal only allows investigation of main features of their panel of cell lines, small molecules and corresponding targets, future updates will provide users with advanced clustering tools to investigate the grouping of compounds based on their growth inhibitory effects (Paul Clemons, ‘personal communication').

#### Cancer Target Discovery And Development

Cancer Target Discovery And Development (CTD2) provides a data portal (https://ctd2.nci.nih.gov/dataPortal/) to cell line experiments produced by members of this research network (including CTRP), each providing different types of data for partially overlapping cancer cell lines. These data include DNA sequencing, gene expression microarrays, comparative genomic hybridization and reverse-phase protein lysate microarrays (RPPA), as well as cytotoxicity screening and perturbation-based screening (e.g. small interfering RNA library screening). Some individual data sets provide multi-assay data [28], and additional depth of integrative analysis of data from genomic profiling and perturbation-based screenings could be gained by combining complementary assays performed on the same cell lines by different laboratories. However, the data formats (e.g. soft,.txt and others) and cell line names (e.g. LN229 versus LN-229) are not standardized, so additional curation is required to integrate data sets originating from the different laboratories.

### Genetic perturbations

#### Project A chilles

Project Achilles provides data sets to identify and catalog genetic vulnerabilities across 216 genomically characterized human cancer cell lines (http://www.broadinstitute.org/achilles). The project uses 54 020 genome-wide genetic perturbation reagents (shRNAs or cas9/sgRNAs) to silence or knock-out around 11 000 individual genes and identify those genes that affect cell proliferation and/or viability. When functional data are integrated with information obtained by cancer genomes, it is possible to reveal lineage-specific dependencies across a wide range of cancers [29]. The project also provides relative abundance assays for shRNA sequences, correlations of genetic dependencies with cell proliferation and lineage-specific mutations. Although not straightforward, network analysis is useful for integrating these types of multiple assays that provide various vulnerability measurements. Network analysis and other methods [30–32] are useful in cataloging synthetic lethality, i.e. finding minimal combinations of genes whose collective inhibition is lethal. Such vulnerability studies provide targets for therapy, and can be performed via integrating publicly available data.

#### CCBR-OICR Lentiviral Technology Cancer

CCBR-OICR Lentiviral Technology (COLT)-Cancer [33] is a Web interface for shRNA screens across multiple cancer cell lines (http://dpsc.ccbr.utoronto.ca/cancer/). The database provides shRNA dropout signature profiles, based on a lentiviral shRNA screening library (78 432 shRNAs) targeting ∼16 000 genes in over 70 cell lines from breast, pancreatic and ovarian cancer. Both shRNA- and gene-activity rank profiles are computed using a developed scoring method to assess their performance in every experiment. Users can assess the activity performance via gene-centric searches, as well as conduct ‘cross cell-line' queries. Compared with the existing repositories of RNAi screens for mammalian cell lines, which support the design of RNAi screens and RNAi analysis of single systems, COLT-Cancer facilitates comparison of essential genes across multiple cell lines. Accordingly, this promises seamless integration of genetic profile data with cancer genomic information, which serves to aid in the identification and development of prognostics and therapeutics for cancer.

### Combination of drug and genetic perturbations

#### Library of Integrated Network-based Cellular Signals

The NIH Library of Integrated Network-based Cellular Signatures (LINCS) project (http://www.lincsproject.org/) has assembled 44 assays for approximately 5943 perturbagens (perturbing agents) across 356 cell lines from six centers, to catalog changes in gene expression and other cellular processes that occur when cells are exposed to a variety of perturbing agents. As part of LINCS, the Connectivity Map project investigates effects of drug compounds on the transcriptional state of cell lines [34, 35]. The most recent version of the Connectivity Map is the L1000 data set, where expression of 1000 ‘landmark genes' [36] is measured for up to 77 cell lines, perturbed by 20 431 compounds. The remaining transcriptome is estimated from a computational model based on thousands of gene expression from the Gene Expression Omnibus. In addition to small molecules and FDA-approved drugs, single gene knockdown and overexpression are available for 5806 genetic perturbations. The LINCS Canvas Browser [37] allows querying, browsing and interrogating of LINCS data. The idea behind the Connectivity Map and the LINCS project is to develop a tool to accelerate the drug discovery process.

### Population genomics

Population genomics examines the genomic variation within and among populations. The most common tool used has been the SNP array, but current efforts include DNA sequencing, RNA sequencing and other data types.

### 1000 Genomes Project

The 1000 Genomes Project aims to provide a comprehensive resource for human genetic variants across the population. Although deep sequencing (which requires 28× coverage) is still expensive to recover the complete genotype of each sample, the Project provides 4× coverage of the genomic regions, enough to identify variants with frequencies as low as 1% in the population. A validated haplomap (a catalog of genetic variants) of 38 million SNPs (98% of accessible SNPs) and 1.4 million short insertions and deletions, among others using this low-coverage whole genome and exome sequencing, have been reported by the 1000 Genomes Project Consortium [38]. The project also provides publicly available expression data (RNA sequencing and expression arrays), which can be analyzed to determine whether genetic variants are associated with changes in expression.

### dbSNP

NCBI’s dbSNP allows users to deposit short genetic variations including SNPs in the Variant Call Format with an asserted position of the variant, thereby providing accuracy in variant mapping [37]. Data from the 1000 Genomes Project get submitted to dbSNP, and longer structural variants get submitted to the Database of Genomic Variants archive, which accounts for variations ranging from tens to millions of base pairs, including insertions, deletions, inversions, translocations and locus copy number changes. Although dbSNP contains a broad collection of SNPs from multiple sources, additional curation and integration of mRNA transcripts is required to perform functional analyses of how the location of the variations affect phenotypic changes such as metabolism and cell signaling. Because dbSNP was developed to complement GenBank, it contains nucleotide sequences from any organism. The human data in dbSNP include submissions from the SNP Consortium, variations mined from genome sequencing as part of the human genome project and individual laboratory contributions of variations in specific genes, mRNAs, Expressed Sequence Tags or genomic regions.

### Exome sequencing projects

Whole-exome sequencing (WES) has been widely adopted, and several initiatives have emerged to structure and gather the large number of profiled samples. The most prominent ones are the National Heart, Lung and Blood Institute Exome Sequencing Project (http://evs.gs.washington.edu/EVS), which has WES data for 6500 individuals diagnosed with heart, lung and blood disorders, and the Exome Aggregation Consortium (http://exac.broadinstitute.org), which has WES data for 60 706 unrelated individuals sequenced as part of various disease-specific and population genetic studies. These initiatives provide an unprecedented depth to characterize rare variants located in exons.

### British birth cohort study

The European Genome-phenome Archive (EGA) is the European version of the service provided by dbSNP. The British birth cohort study archive is set up to provide 10 TB of data from 1 million loci taken from 100 000 individuals from the Genomics Englands’ Genome 10K project. In addition, the archive handles both restricted patient data and freely available data after phenotypic information of the individuals are removed. A number of studies have been published since the initiation of EGA, including genome-wide association study (GWAS) (http://www.gwascentral.org/), cancer genomics (https://ocg.cancer.gov/) and whole genome sequencing (http://www.illumina.com/applications/sequencing/dna_sequencing/whole_genome_sequencing.html), in addition to genotype and expression analyses. These data have been used in key discoveries in common diseases, see for example [39–41].

### Clinical covariates in consortial oncology projects

Omics data intended for the study of disease are far less useful if not coupled with comprehensive clinical records. Yet, standardizing and digitizing clinical data remains a challenge that only in the recent years has started to be addressed [42]. Cancer consortium projects, such as TCGA or ICGC, can enforce standards for the integration of clinical and molecular data produced within the consortium. However, even with such focused efforts, it has been reported that much of the ICGC molecular data lack key clinical information [43]. Storing molecular and clinical data also requires maintaining patient's anonymity and matching patient's consent to the use of their data. The NCBI dbGaP database of genotypes and phenotypes [44] and EGA [45] are two working instances of technological platforms meeting those requirements.

## Regulatory genomics

### Encyclopedia of DNA Elements

The Encyclopedia of DNA Elements (ENCODE) Consortium is an international collaboration of research groups funded by the NHGRI. The ENCODE project was launched to reveal how genetic instructions are read on a global, genome-wide scale [46]. As a result of the ENCODE effort, a detailed picture of human genome organization is emerging which includes the mapping of transcribed regions [47], DNA binding of transcription factors [48], and the structure and modifications of chromatin states [49]. Collectively, the project surveys the landscape of the *Homo* *sapiens* and *Mus* *musculus* genomes using over 20 high-throughput genomic assays in >350 different cell and tissue types, resulting in over 3000 data sets [50, 51]. This information is informative to both basic and disease-related human biology [52]. The ENCODE portal is the primary repository to access, view and download all data generated by the ENCODE consortium (http://www.encodeproject.org). Direct interaction with the ENCODE Data Coordination Centre database can be performed by the ENCODE REST API. Furthermore, a number of software tools help users to use the ENCODE data in their own analyses (https://www.encodeproject.org/software). Besides providing users with an unprecedented amount of data from state-of-the-art functional genome-wide assays, the ENCODE project has set the stage for developing computational approaches to correlate multiple data types and derive quantitative models of gene expression regulation [49].

The ENCODE project has been used as a resource for common diseases other than cancer, including insulin resistance [53] and kidney disease [54]. GWAS profiling has been used to identify dysregulated genes in autoimmune disease [55]. The common theme of these studies has been to connect disease-associated genes with their controlling regulatory elements.

### Functional Annotation of Mammalian Genomes 5

The Functional Annotation of Mammalian Genomes 5 (FANTOM5) (http://fantom.gsc.riken.jp/5/) project aims at building transcriptional regulatory models for every primary cell type that makes up a human. The RIKEN-led FANTOM5 consortium systematically investigates the sets of genes used in virtually all cell types across the human body, and the genomic regions which determine where the genes are read from. What emerges from applying Cap Analysis of Gene Expression to the majority of mammalian primary cell types and a selection of cancer cell lines and tissues is a fined and context-specific map of sets of active transcripts, transcription factors, promoters and enhancers [56, 57]. More recently, a comprehensive analysis of RNA expression in 19 human time courses showed that enhancer transcription is the earliest event in successive waves of transcriptional changes when cells undergo phenotype changes such as differentiation into specialized cell types [58]. To help wet-bench and computational users to examine the diverse and large number of samples, the FANTOM5 consortium assembled the FANTOM Five Sample Ontology leveraging on the existing basic ontologies regarding cell types (CL), anatomical systems (UBERON) and diseases (DOID). Primary data visualization system for FANTOM5 is the ZENBU genome browser and analysis system. All data generated by the FANTOM5 project can also be accessed by using a semantic catalog of samples, transcription initiation and regulators or by sub-setting data of interest through the Table Extraction Tool [59].

### Ensembl Regulatory Build

The Ensembl Regulatory Build aims at providing an up-to-date and systematic overview of regulatory elements by integrating the large amounts of valuable data released into the public domain from the aforementioned projects as well as from medium-scale studies [60].

## Microarray repositories

### Gene Expression Omnibus

**Figure 3.**
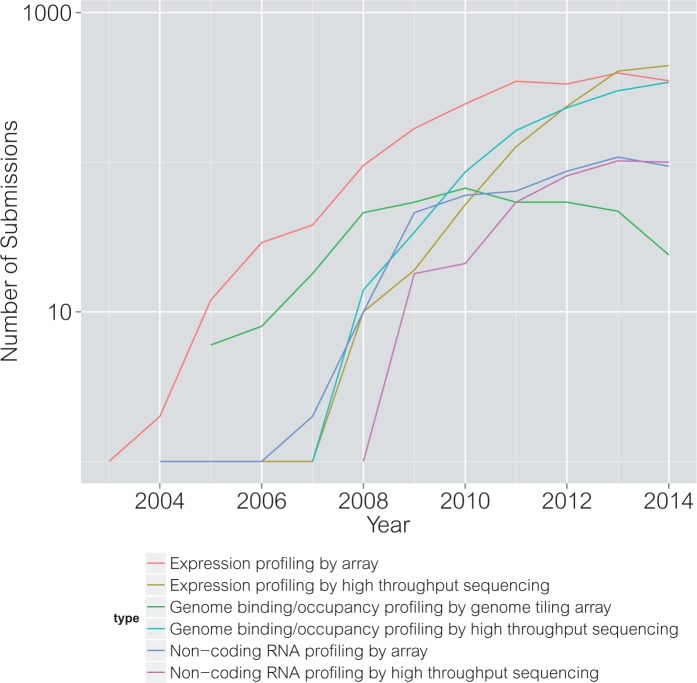
The growth of multi-assay genomic data sets in GEO. The GEOmetadb Bioconductor package [61] was used to identify all GEO series providing two or more data types. Using this subset of GEO series, the number of each of these data types was counted per year, and the six most common types are shown. The majority of multi-assay data set in GEO include expression profiling, noncoding RNA profiling and/or genome binding/occupancy profiling, each by array or high-throughput sequencing, with the number of sequencing experiments catching up to arrays in 2014.

Multi-assay genomic experiments are increasingly within reach of individual laboratories, and GEO is the primary database where data from these experiments are shared publicly. Data from GEO can be challenging to use because (1) data sets are challenging to find among the >57 000 data series and 14 000 technological platforms provided at time of writing, and (2) important aspects of data sets such as gene identifiers and clinical annotations are not consistent between data sets. The first challenge can be approached using the ‘GEOmetadb' Bioconductor package [61], which provides a SQLite database of all GEO metadata that can be accessed through SQL queries or simplified interfaces such as the ‘dplyr' library [62]. We used ‘GEOmetadb' to identify all 3910 GEO series providing more than one genomic data type (Table 3), and plotted the number of the six most common data types appearing among these series each year since the first series with multiple data types appeared in 2003 (Figure 3). Code for these analyses are provided at https://github.com/seandavi/MultiplatformGEOSurvey. Data sets identified by this approach can be downloaded in uncurated form to the Bioconductor environment [63] using ‘GEOquery' [64].

**Table 3.** Multi-assay data sets in the GEO as of April, 2015. Data types provided by every GEO series were queried using the ‘GEOmetadb' Bioconductor package [61]

Number of data types	Number of GEO series
1	53 066
2	3382
3	456
4	64
5	8

### ArrayExpress

ArrayExpress (https://www.ebi.ac.uk/arrayexpress/) [65] is the European counterpart of GEO and also a major resource for experimental data from individual laboratories. ArrayExpress lacks an equivalent of ‘GEOmetadb' but does have an interface to Bioconductor [66].

### Georgetown Database of Cancer Plus other diseases

The Georgetown Database of Cancer (G-DOC) Plus (https://gdoc.georgetown.edu/gdoc/) provides ‘omics' data from 59 clinical studies covering 10 201 patients, including 10 cancer types and other diseases such as dementia, muscular dystropy and wound healing. The repository provides individual patient data including demographics, clinical outcome, and tumor pathology. The data in G-DOC Plus are uniformly processed in Bioconductor, and then uploaded to the central database.

### Expression Quantitative Trait Loci studies

Expression Quantitative Trait Loci (eQTL) studies are possibly the most common existing examples of multi-assay genomic analysis. The aim of eQTL studies, where mRNA expression levels are considered as quantitative traits, is the identification of genetic variants affecting gene regulation [67]; to this end, eQTL studies integrate genotypic and expression data. In human studies, the genotype portion of eQTL experiments is often not made publicly available because of privacy concerns. The Genotype-Tissue Expression project (http://www.gtexportal.org) [68, 69] is to our knowledge the most comprehensive data resource for gene expression and genotype across multiple human tissues, and provides genotype data to verified researchers. GTEx and other major gene regulation catalogs were recently summarized [70].

### STATegra

STATegra (http://www.stategra.eu) profiles a well-established model of cell-system B-cell progenitor differentiation including Hardy fractions Fr.C' to Fr.D (B3, [71]). Cells are assayed with strand-specific RNA-seq, ChIP-seq, RRBS-seq, DNase-seq, microRNA-seq, proteomics and metabolomics, to provide a controlled system for investigating the different regulatory mechanisms of mRNA during a differentiation process. In contrast to TCGA, where many data types are considered for different cancers, STATegra data provide a more detailed genomic profiling over a limited number of biological replications. STATegra can be considered as a snapshot of future projects where many omics would be available for the same system. STATegra data will become public by the second half of 2015, and will provide a test case for data management and integrative analysis methods for extensive multi-assay profiling.

### Genomic data sets for single-disease systems

**Figure 4.**
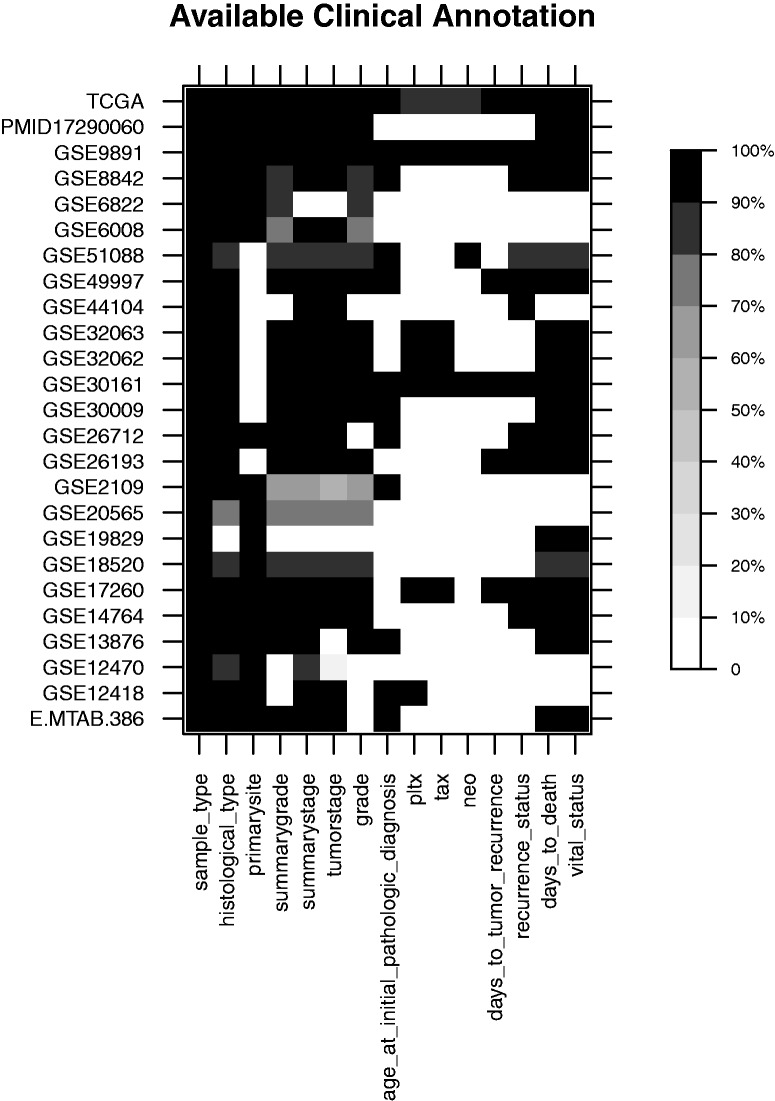
Overview of clinical annotation in the curatedOvarian data package. Clinicopathological characteristics of patients (columns) are represented across 25 gene expression data sets (rows). For each data set, the percentage of patients in that data set that are annotated by a certain clinical characteristic is represented. Patient treatment by platin, taxol or neoadjuvant therapy is presented as pltx, tax and neo, respectively.

Several highly curated ‘ExperimentData' packages in Bioconductor provide an easy and focused point of entry into integrative genomic data analysis for single disease systems. The curatedOvarianData package [72], for example, is a compendium of 25 highly annotated gene expression data sets that encompasses over 3000 ovarian cancer clinically annotated gene and microRNA expression profiles. These samples have been collated across multiple studies from GEO, EBI Array Express, TCGA, as well as individually archived datasets. Key clinical annotations such as stage, grade, primary site, and outcome are present across most data sets, but other variables such as patient treatment information are mostly absent (Figure 4). The curatedOvarianData package serves as a prime example of how harmonization of public data sets can enable new hypothesis testing and development of statistical methodology.

## Tools for integrative genomic data analysis

Biological molecules are regulated at the transcriptional, translational, and post-translational level. Integrating different molecular data may increase the power to more comprehensively interrogate and understand the physiological system, but novel methodologies are required to integrate these complex data sets. We survey available tools in several categories before concluding with the still-existing gaps in these tools.

### Interactive visualization tools

We classify visualization tools by the usage of genomic coordinates, heat maps, and networks. Extensive overviews of visualization tools are available [73], and this section only briefly surveys a few of the most popular alternatives.

Genome browsers such as the UCSC Cancer Genomics Browser [74] and the Savant Genome Browser [75] allow data visualization by genomic location, with addition of annotations and data types as additional tracks. The Integrative Genomics Viewer [76] additionally enables inspection of specific cancer genomic loci or the general overview of genomic alterations, the exploration of cause–effect relationships between various alteration types. Most recently, the Epiviz genome browser [77] provides both a web-based interface and full integration with Bioconductor and its core data classes through the ‘Epivizr' package. Visualization tools tied to genomic coordinates can be troublesome in cancer genomes containing extensive rearrangements, and when attempting to integrate data types not associated with genomic coordinates. Alternate visualization tools such as circle plots [78, 79] and canonical correlation analysis methods are useful to circumvent the challenges posed by rearrangements.

Several online resources preload data from TCGA, ICGC and GEO, and generate heatmaps and other visualizations from these data. cBioPortal (http://www.cbioportal.org) [11] is a popular Web interface, that presents results of integrative analyses, visualizations, and selective downloads of TCGA and other cancer data sets. Direct programmatic queries to the cBioPortal cancer genomics data server (CGDS) is available using a REST API, Matlab, and an R package (CGDS-R). IntOgen (http://www.intogen.org) [80, 81] provides publicly available cancer genomic data, as well as drug–protein interactions, to provide a point-and-click interface to the integrative analysis of these data with a focus on visualization of somatic mutations. Caleydo StratomeX (http://caleydo.github.io/) [82] provides heatmaps, stratification by clustering of multiple data types and by clinico-pathological parameters, Kaplan-Meier plots, differential expression analysis, and gene set enrichment analysis for TCGA data. Gitools [83] generates interactive heatmaps of TCGA as well as IntOgen mutation data sets. GENE-E (unpublished, http://www.broadinstitute.org/cancer/software/GENE-E) is yet another tool focused on integrative genomic heatmaps, but does not focus on preloaded data. The Regulome Explorer (http://explorer.cancerregulome.org/all_pairs/) visualizes relationships between pairs of genomic measurements in an interactive circular link diagram.

### Data analysis and methodology development in Bioconductor

The above tools provide predetermined analyses and visualizations, as opposed to software for open-ended exploratory data analysis or development of integrative methodology. The Bioconductor project [63] provides one of the most widely used, most flexible and mature open source environments for such purposes. Bioconductor provides statistical software for preprocessing, normalization, analysis, integration, and visualization of numerous molecular data types including exome, RNAseq, methylation, microRNA, proteomics data. It is widely considered the *de* *facto* statistical analysis suite for genomics data. The major data classes are ‘GenomicRanges' and ‘SummarizedExperiment' for processed range-based data [84], and ‘ExpressionSet' for data represented by features such as gene or microarray probeset identifiers [85]. These classes greatly simplify record-keeping and integrative analysis of a single data type with patient characteristics or other phenotypes. ‘GenomicRanges' and its associated ‘IRanges' algebra assist in the integration of multiple genome-anchored data types through built-in operations to find intersections, unions, flanking regions, etc of genomic ranges. However, base data classes for multi-assay genomic experiments are still lacking.

Bioconductor enables integration with annotation resources such as gene models, regulatory interactions, and maps between alternative genomic feature identifiers. Organism-oriented, systems biology-oriented, and gene and transcript-oriented annotation resources have been summarized (http://genomicsclass.github.io/book/pages/annoCheat.html). Recently, major annotation resources from ENCODE and the UCSC Genome Browser are redistributed within Bioconductor via ‘AnnotationHub'.

We classify data integration methodologies under three categories: exploratory data analysis, network analysis and supervised meta-analysis. Exploratory data analysis enables one to identify the major patterns in the data, including potential issues such as batch effects [86] and outliers. Multivariate extensions of principal component analysis (PCA) and clustering analysis are useful tools to understand basic data structure, inter-data set and intra-data set correlations. Some of the R software available in this category are PMA [97], made4 [87], MCIA [88], RGCCA [89]. Network analysis has been applied for the integration of multiple data types. A common approach is to combine an interaction network with molecular (e.g. genomic, transcriptomic and proteomic) profiles reflecting condition-specific nodes activities or interaction strengths [91–92, 95]. In supervised meta-analysis, the aim is to refine findings based on one data type with additional analysis of further omics data obtained from the same set of samples. Some of the available software in this category are CNAmet [93], Rtopper [94], iClusterPlus [95] and the STATegra Bioconductor package. We note that these methods fall short of full systems biology data integration, as they do not, for example, incorporate known regulatory relationships between microRNA, RNA-binding protein or transcription factors, with gene expression. While a thorough review of these resources is beyond the scope of this review, we summarize some relevant R/Bioconductor packages in Table 4, and refer readers to [96] for a comprehensive review of genomic data analysis in Bioconductor.

**Table 4.** R packages for integrative data analysis

R/bioconductor package name	Description	Repository
PMA [97]	Penalized multivariate analysis (sparse CCA, PCA)	CRAN
mixOmics [98]	rCCA, sPLS	CRAN
	sPLS-DA	
	rGCCA	
made4 [87]	Coinertia analysis	Bioconductor
MCIA [88]	Multi-CIA	Bioconductor
RGCCA [89]	rGCCA, sparse GCCA for multi-block data analysis	CRAN
CNAmet [93]	Signal-to-noise ratio statistic, permutation test	csbi.ltdk.helsinki.fi/CNAmet/
Rtopper [94]	Gene set enrichment	Bioconductor
iClusterPlus [95]	Joint latent variable regression model	Bioconductor
STATegra (www.stategra.eu)	PCA, clustering	Bioconductor

## Discussion

The data resources and toolsets outlined in this review are used by disparate research communities, but integration across data sets and data types remains limited. A significant barrier to better utilization of these free resources is simply finding the right data or tool among an overwhelming amount of loosely organized information. The US NIH have recognized this barrier and announced plans to develop a Data Commons (https://pebourne.wordpress.com/2014/10/07/the-commons/) that will serve as a metadata repository for data and software resources. This review is written with a similar but more focused intent, providing a much more focused snapshot of a resources for multi-assay genomic data on human disease.

Sufficient computing infrastructure, and the cost of moving large data sets to local infrastructure to compute on these data sets can be an additional barrier. The Cancer Genomics Cloud Pilots of the US NCI (https://cbiit.nci.nih.gov/ncip/nci-cancer-genomics-cloud-pilots) is one experiment in addressing some of these infrastructural limitations by co-location of computing infrastructure, data and software, and commercial options are also appearing. Flexible computing approaches, which maximize use of whatever resources are available, are needed. For example, expression and phenotype data on samples may be available as tables on local disk, while high-resolution genotype data on the samples may be resident in cloud-based storage, and a local cluster may have a large number of compute nodes with modest memory volumes available to support machine learning. Highly generic workflow specification is needed to support derivation, from diverse sources and storage modalities, of data subsets that are suitable inputs to statistical algorithms. Distributed implementations of statistical algorithms, with scalable memory footprints, must also be readily usable by subject-matter specialists. The Bioconductor project has experienced substantial recent advances in such scalable computing by streamlining programming idioms for embarrassingly parallel computation (BiocParallel package), creating virtual machine images to simplify endowment of clusters with strong numerical and inferential toolkits (Bioconductor AMI) and emphasizing scalable data flow architecture both for assay interrogation (record yield prescriptions for BAM archive references in ‘Rsamtools') and statistical computation (scatter-gather accumulation of sufficient statistics in the ‘parglm' package). There remain significant needs for input on strategy selection for data storage, harvesting, and analysis processes.

Although abundant multi-assay genomic data resources exist, software tools for their analysis are still severely lacking. Available tools focus primarily on data acquisition and standard visualization, and provide some common prespecified analyses. True systems biology integration of different layers of genomic data still requires custom coding and substantial bioinformatics effort. The Bioconductor project comes closest to providing an environment for systems biology integration with arbitrarily flexible statistical analysis and visualization, but important developments are still needed. The creation of multi-assay containers will enable advanced quality control checks through the assessment of known regulators effects on targets at multiple levels such as DNA methylation versus mRNA transcription or DNA copy number versus mRNA transcription. Fully integrative analysis requires linking data types through annotation. A few possible examples include linking microRNA and their targets, associating exons with transcripts or transcripts with genomic locations of respective genes or with other transcripts in known pathways. All these annotations exist within the Bioconductor environment, but facilities allowing straightforward integration of them remains an area of active current development.

Key Points

- Although abundant multi-assay genomic data resources exist, software tools for their analysis are still in early stages.
- Available tools focus primarily on data acquisition and visualization through genome browsers or heatmaps, and providing some common prespecified analyses.
- Significant efforts are still needed to streamline systems biology integration of different layers of genomic data, and to integrate results from the major data-generating projects.

## Supplementary Data

Supplementary data are available online at http://bib.oxfordjournals.org/.

## Funding

The authors’ work was funded by the National Cancer Institute [U24CA180996 to MM], the National Institute on Minority Health and Health Disparities [MD007599 to LW] and the NCI Intramural Research Program [SD] of the National Institutes of Health. Spanish MINECO grant TIN2011-22826 to RC. BHK was supported by the Gattuso Slaight Personalized Cancer Medicine Fund at Princess Margaret Cancer Centre. ZS was supported by the Cancer Research Society (Canada). DMAG was supported by the Brain Canada-CIBC Brain Cancer Research Training Award AR was supported by the Biotechnology start-up project of the University of Trento. AC was supported by National Cancer Institute 1U19 AI111224-01 and the Assistant Secretary of Defense Health Program, through the Breast Cancer Research Program, Award No. W81XWH-15-1-0013.

## Supplementary Material

Supplementary Data

## References

[1] Gomez-Cabrero D Abugessaisa I Maier D Data integration in the era of omics: current and future challenges. BMC Syst Biol 2014;8 (Suppl 2):I1. 25032990 10.1186/1752-0509-8-S2-I1 PMC4101704

[2] International Cancer Genome Consortium, Hudson TJ Anderson W International network of cancer genome projects. Nature 2010;464:993–8. 20393554 10.1038/nature08987 PMC2902243

[3] Barrett T Wilhite SE Ledoux P NCBI GEO: archive for functional genomics data sets–update. Nucleic Acids Res 2013;41:D991–5. 23193258 10.1093/nar/gks1193 PMC3531084

[4] Kolesnikov N Hastings E Keays M ArrayExpress update-simplifying data submissions. Nucleic Acids Res 2015;43:D1113–6. 25361974 10.1093/nar/gku1057 PMC4383899

[5] Hoadley KA Yau C Wolf DM Multiplatform analysis of 12 cancer types reveals molecular classification within and across tissues of origin. Cell 2014;158:929–44. 25109877 10.1016/j.cell.2014.06.049 PMC4152462

[6] Zhang B Wang J Wang X Proteogenomic characterization of human colon and rectal cancer. Nature 2014;513:382–7. 25043054 10.1038/nature13438 PMC4249766

[7] Yuan Y Van Allen EM Omberg L Assessing the clinical utility of cancer genomic and proteomic data across tumor types. Nat Biotechnol 2014;32:644–52. 24952901 10.1038/nbt.2940 PMC4102885

[8] Samur MK RTCGAToolbox: a new tool for exporting TCGA Firehose data. PLoS One 2014;9:e106397. 25181531 10.1371/journal.pone.0106397 PMC4152273

[9] Broad Institute MIT. Firehose Broad GDAC Download. https://confluence.broadinstitute.org/display/GDAC/Download

[10] Saleem M Padmanabhuni SS Ngomo A-CN Linked Cancer Genome Atlas Database. In: Proceedings of the 9th International Conference on Semantic Systems 2013, ACM, New York, NY, USA, pp. 129–34.

[11] Gao J Aksoy BA Dogrusoz U Integrative analysis of complex cancer genomics and clinical profiles using the cBioPortal. Sci Signal 2013;6:l1. 10.1126/scisignal.2004088 PMC4160307 23550210

[12] Cerami E Gao J Dogrusoz U The cBio cancer genomics portal: an open platform for exploring multidimensional cancer genomics data. Cancer Discov 2012;2:401–4. 22588877 10.1158/2159-8290.CD-12-0095 PMC3956037

[13] Wilks C Cline MS Weiler E The Cancer Genomics Hub (CGHub): overcoming cancer through the power of torrential data. Database 2014;2014:bau093. 25267794 10.1093/database/bau093 PMC4178372

[14] Zhu Y Qiu P Ji Y TCGA-assembler: open-source software for retrieving and processing TCGA data. Nat Methods 2014;11:599–600 24874569 10.1038/nmeth.2956 PMC4387197

[15] Bot B. Sage Bionetworks: Introduction to the Synapse Client. https://www.synapse.org/#!Wiki:syn1834618/ENTITY

[16] National Cancer Institute. The Cancer Genome Atlas: Data Portal. https://tcga-data.nci.nih.gov/tcga/tcgaDownload.jsp

[17] The Cancer Imaging Archive. About The Cancer Imaging Archive (TCIA). http://www.cancerimagingarchive.net/about-the-cancer-imaging-archive-tcia/

[18] Curtis C Shah SP Chin S-F The genomic and transcriptomic architecture of 2,000 breast tumours reveals novel subgroups. Nature 2012;486:346–52. 22522925 10.1038/nature10983 PMC3440846

[19] Shoemaker RH The NCI60 human tumour cell line anticancer drug screen. Nat Rev Cancer 2006;6:813–23. 16990858 10.1038/nrc1951

[20] Reinhold WC Sunshine M Liu H CellMiner: a web-based suite of genomic and pharmacologic tools to explore transcript and drug patterns in the NCI-60 cell line set. Cancer Res 2012;72:3499–511. 22802077 10.1158/0008-5472.CAN-12-1370 PMC3399763

[21] Bussey KJ Chin K Lababidi S Integrating data on DNA copy number with gene expression levels and drug sensitivities in the NCI-60 cell line panel. Mol Cancer Ther 2006;5:853–67. 16648555 10.1158/1535-7163.MCT-05-0155 PMC2733874

[22] Varma S Pommier Y Sunshine M High resolution copy number variation data in the NCI-60 cancer cell lines from whole genome microarrays accessible through CellMiner. PLoS One 2014;9:e92047. 24670534 10.1371/journal.pone.0092047 PMC3966786

[23] Yu M Selvaraj SK Liang-Chu MMY A resource for cell line authentication, annotation and quality control. Nature 2015;520:307–11. 25877200 10.1038/nature14397

[24] Barretina J Caponigro G Stransky N The Cancer Cell Line Encyclopedia enables predictive modelling of anticancer drug sensitivity. Nature 2012;483:603–7. 22460905 10.1038/nature11003 PMC3320027

[25] Macconaill LE Garraway LA Clinical implications of the cancer genome. J Clin Oncol 2010;28:5219–28. 20975063 10.1200/JCO.2009.27.4944 PMC3020694

[26] Garnett MJ Edelman EJ Heidorn SJ Systematic identification of genomic markers of drug sensitivity in cancer cells. Nature 2012;483:570–5. 22460902 10.1038/nature11005 PMC3349233

[27] Basu A Bodycombe NE Cheah JH An interactive resource to identify cancer genetic and lineage dependencies targeted by small molecules. Cell 2013;154:1151–61. 23993102 10.1016/j.cell.2013.08.003 PMC3954635

[28] Cheung HW Cowley GS Weir BA Systematic investigation of genetic vulnerabilities across cancer cell lines reveals lineage-specific dependencies in ovarian cancer. Proc Natl Acad Sci USA 2011;108:12372–7. 21746896 10.1073/pnas.1109363108 PMC3145679

[29] Cowley GS Weir BA Vazquez F Parallel genome-scale loss of function screens in 216 cancer cell lines for the identification of context-specific genetic dependencies. Scientific Data 2014;1:140035. 25984343 10.1038/sdata.2014.35 PMC4432652

[30] Jerby-Arnon L Pfetzer N Waldman YY Predicting cancer-specific vulnerability via data-driven detection of synthetic lethality. Cell 2014;158:1199–209. 25171417 10.1016/j.cell.2014.07.027

[31] Aksoy BA Demir E Babur Ö Prediction of individualized therapeutic vulnerabilities in cancer from genomic profiles. Bioinformatics 2014;30:2051–9. 24665131 10.1093/bioinformatics/btu164 PMC4080742

[32] Nijhawan D Zack TI Ren Y Cancer vulnerabilities unveiled by genomic loss. Cell 2012;150:842–54. 22901813 10.1016/j.cell.2012.07.023 PMC3429351

[33] Koh JLY Brown KR Sayad A COLT-Cancer: functional genetic screening resource for essential genes in human cancer cell lines. Nucleic Acids Res 2012;40:D957–63. 22102578 10.1093/nar/gkr959 PMC3245009

[34] Lamb J Crawford ED Peck D The Connectivity Map: using gene-expression signatures to connect small molecules, genes, and disease. Science 2006;313:1929–35. 17008526 10.1126/science.1132939

[35] NIH, Broad Institute. The LINCS Connectivity Map Project. The LINCS Connectivity Map Project 2013;**19**:63–74.

[36] Peck D Crawford ED Ross KN A method for high-throughput gene expression signature analysis. Genome Biol 2006;7:R61. 16859521 10.1186/gb-2006-7-7-r61 PMC1779561

[37] Duan Q Flynn C Niepel M LINCS Canvas Browser: interactive web app to query, browse and interrogate LINCS L1000 gene expression signatures. Nucleic Acids Res 2014;42:W449–60. 24906883 10.1093/nar/gku476 PMC4086130

[38] Genomes Project Consortium, Abecasis GR Auton A An integrated map of genetic variation from 1,092 human genomes. Nature 2012;491:56–65. 23128226 10.1038/nature11632 PMC3498066

[39] Jefferis BJMH Power C Hertzman C Birth weight, childhood socioeconomic environment, and cognitive development in the 1958 British birth cohort study. BMJ 2002;325:305. 12169505 10.1136/bmj.325.7359.305 PMC117769

[40] Hyppönen E Power C Vitamin D status and glucose homeostasis in the 1958 British birth cohort: the role of obesity. Diabetes Care 2006;29:2244–6. 17003300 10.2337/dc06-0946

[41] Viner RM Cole TJ Who changes body mass between adolescence and adulthood? Factors predicting change in BMI between 16 year and 30 years in the 1970 British Birth Cohort. Int J Obes 2006;30:1368–74. 10.1038/sj.ijo.0803183 16552412

[42] Kaushal R Hripcsak G Ascheim DD Changing the research landscape: the New York City Clinical Data Research Network. J Am Med Inform Assoc 2014;21:587–90. 24821739 10.1136/amiajnl-2014-002764 PMC4078297

[43] Rubin MA Health: make precision medicine work for cancer care. Nature 2015;520:290–1. 25877189 10.1038/520290a

[44] Mailman MD Feolo M Jin Y The NCBI dbGaP database of genotypes and phenotypes. Nat Genet 2007;39:1181–6. 17898773 10.1038/ng1007-1181 PMC2031016

[45] Lappalainen I Almeida-King J Kumanduri V The European Genome-phenome Archive of human data consented for biomedical research. Nat Genet 2015;47:692–5. 26111507 10.1038/ng.3312 PMC5426533

[46] ENCODE Project Consortium. An integrated encyclopedia of DNA elements in the human genome. Nature 2012;489:57–74. 22955616 10.1038/nature11247 PMC3439153

[47] Djebali S Davis CA Merkel A Landscape of transcription in human cells. Nature 2012;489:101–8. 22955620 10.1038/nature11233 PMC3684276

[48] Neph S Vierstra J Stergachis AB An expansive human regulatory lexicon encoded in transcription factor footprints. Nature 2012;489:83–90. 22955618 10.1038/nature11212 PMC3736582

[49] Thurman RE Rynes E Humbert R The accessible chromatin landscape of the human genome. Nature 2012;489:75–82. 22955617 10.1038/nature11232 PMC3721348

[50] Mouse ENCODE Consortium, Stamatoyannopoulos JA Snyder M An encyclopedia of mouse DNA elements (Mouse ENCODE). Genome Biol 2012;13:418. 22889292 10.1186/gb-2012-13-8-418 PMC3491367

[51] Boyle AP Araya CL Brdlik C Comparative analysis of regulatory information and circuits across distant species. Nature 2014;512:453–6. 25164757 10.1038/nature13668 PMC4336544

[52] Michailidou K Beesley J Lindstrom S Genome-wide association analysis of more than 120,000 individuals identifies 15 new susceptibility loci for breast cancer. Nat Genet 2015;47:373–80. 25751625 10.1038/ng.3242 PMC4549775

[53] Lo KA Labadorf A Kennedy NJ Analysis of in vitro insulin-resistance models and their physiological relevance to in vivo diet-induced adipose insulin resistance. Cell Rep 2013;5:259–70. 24095730 10.1016/j.celrep.2013.08.039 PMC3874466

[54] Susztak K Understanding the epigenetic syntax for the genetic alphabet in the kidney. J Am Soc Nephrol 2014;25:10–17. 24179169 10.1681/ASN.2013050461 PMC3871782

[55] Lettre G Rioux JD Autoimmune diseases: insights from genome-wide association studies. Hum Mol Genet 2008;17:R116–21. 18852199 10.1093/hmg/ddn246 PMC2782355

[56] Andersson R Gebhard C Miguel-Escalada I An atlas of active enhancers across human cell types and tissues. Nature 2014;507:455–61. 24670763 10.1038/nature12787 PMC5215096

[57] FANTOM Consortium and the RIKEN PMI and CLST (DGT), Forrest ARR Kawaji H A promoter-level mammalian expression atlas. Nature 2014;507:462–70. 24670764 10.1038/nature13182 PMC4529748

[58] Arner E Daub CO Vitting-Seerup K Gene regulation. Transcribed enhancers lead waves of coordinated transcription in transitioning mammalian cells. Science 2015;347:1010–14. 25678556 10.1126/science.1259418 PMC4681433

[59] Lizio M Harshbarger J Shimoji H Gateways to the FANTOM5 promoter level mammalian expression atlas. Genome Biol 2015;16:22. 25723102 10.1186/s13059-014-0560-6 PMC4310165

[60] Zerbino DR Wilder SP Johnson N The ensembl regulatory build. Genome Biol 2015;16:56. 25887522 10.1186/s13059-015-0621-5 PMC4407537

[61] Zhu Y Davis S Stephens R GEOmetadb: powerful alternative search engine for the Gene Expression Omnibus. Bioinformatics 2008;24:2798–800. 18842599 10.1093/bioinformatics/btn520 PMC2639278

[62] Wickham H Francois R dplyr: A Grammar of Data Manipulation. 2015.

[63] Gentleman RC Carey VJ Bates DM Bioconductor: open software development for computational biology and bioinformatics. Genome Biol 2004;5:R80. 15461798 10.1186/gb-2004-5-10-r80 PMC545600

[64] Davis S Meltzer PS GEOquery: a bridge between the Gene Expression Omnibus (GEO) and BioConductor. Bioinformatics 2007;23:1846–7. 17496320 10.1093/bioinformatics/btm254

[65] Parkinson H Kapushesky M Shojatalab M ArrayExpress–a public database of microarray experiments and gene expression profiles. Nucleic Acids Res 2006;35:D747–50. 17132828 10.1093/nar/gkl995 PMC1716725

[66] Kauffmann A Rayner TF Parkinson H Importing ArrayExpress datasets into R/Bioconductor. Bioinformatics 2009;25:2092–4. 19505942 10.1093/bioinformatics/btp354 PMC2723004

[67] Gilad Y Rifkin SA Pritchard JK Revealing the architecture of gene regulation: the promise of eQTL studies. Trends Genet 2008;24:408–15. 18597885 10.1016/j.tig.2008.06.001 PMC2583071

[68] GTEx Consortium. The Genotype-Tissue Expression (GTEx) project. Nat Genet 2013;45:580–5. 23715323 10.1038/ng.2653 PMC4010069

[69] The GTEx Consortium. The Genotype-Tissue Expression (GTEx) pilot analysis: multitissue gene regulation in humans. Science 2015;348:648–60. 25954001 10.1126/science.1262110 PMC4547484

[70] Pennisi E New database links regulatory DNA to its target genes. Science 2015;348:618–19. 25953987 10.1126/science.348.6235.618

[71] Ferreirós-Vidal I Carroll T Taylor B Genome-wide identification of Ikaros targets elucidates its contribution to mouse B-cell lineage specification and pre-B–cell differentiation. Blood 2013;121:1769–82. 23303821 10.1182/blood-2012-08-450114

[72] Ganzfried BF Riester M Haibe-Kains B curatedOvarianData: clinically annotated data for the ovarian cancer transcriptome. Database 2013;2013:bat013. 23550061 10.1093/database/bat013 PMC3625954

[73] Schroeder MP Gonzalez-Perez A Lopez-Bigas N Visualizing multidimensional cancer genomics data. Genome Med 2013;5:9. 23363777 10.1186/gm413 PMC3706894

[74] Goldman M Craft B Swatloski T The UCSC Cancer Genomics Browser: update 2015. Nucleic Acids Res 2015;43:D812–7. 25392408 10.1093/nar/gku1073 PMC4383911

[75] Fiume M Smith EJM Brook A Savant Genome Browser 2: visualization and analysis for population-scale genomics. Nucleic Acids Res 2012;40:W615–21. 22638571 10.1093/nar/gks427 PMC3394255

[76] Thorvaldsdóttir H Robinson JT Mesirov JP Integrative Genomics Viewer (IGV): high-performance genomics data visualization and exploration. Brief Bioinform 2013;14:178–92. 22517427 10.1093/bib/bbs017 PMC3603213

[77] Chelaru F Smith L Goldstein N Epiviz: interactive visual analytics for functional genomics data. Nat Methods 2014;11:938–40. 25086505 10.1038/nmeth.3038 PMC4149593

[78] Krzywinski M Schein J Birol I Circos: an information aesthetic for comparative genomics. Genome Res 2009;19:1639–45. 19541911 10.1101/gr.092759.109 PMC2752132

[79] Gu Z Gu L Eils R circlize Implements and enhances circular visualization in R. Bioinformatics 2014;30:2811–12. 24930139 10.1093/bioinformatics/btu393

[80] Gundem G Perez-Llamas C Jene-Sanz A IntOGen: integration and data mining of multidimensional oncogenomic data. Nat Methods 2010;7:92–3. 20111033 10.1038/nmeth0210-92

[81] Rubio-Perez C Tamborero D Schroeder MP In silico prescription of anticancer drugs to cohorts of 28 tumor types reveals targeting opportunities. Cancer Cell 2015;27:382–96. 25759023 10.1016/j.ccell.2015.02.007

[82] Turkay C Lex A Streit M Characterizing cancer subtypes using dual analysis in Caleydo StratomeX. IEEE Comput. Graph Appl 2014;34:38–47. 24808198 10.1109/MCG.2014.1 PMC4196636

[83] Perez-Llamas C Lopez-Bigas N Gitools: analysis and visualisation of genomic data using interactive heat-maps. PLoS One 2011;6:e19541. 21602921 10.1371/journal.pone.0019541 PMC3094337

[84] Lawrence M Huber W Pagès H Software for computing and annotating genomic ranges. PLoS Comput Biol 2013;9:e1003118. 23950696 10.1371/journal.pcbi.1003118 PMC3738458

[85] Gautier L Cope L Bolstad BM affy–analysis of Affymetrix GeneChip data at the probe level. Bioinformatics 2004;20:307–15. 14960456 10.1093/bioinformatics/btg405

[86] Leek JT Scharpf RB Bravo HC Tackling the widespread and critical impact of batch effects in high-throughput data. Nat Rev Genet 2010;11:733–9. 20838408 10.1038/nrg2825 PMC3880143

[87] Culhane AC Perrière G Higgins DG Cross-platform comparison and visualisation of gene expression data using co-inertia analysis. BMC Bioinformatics 2003;4:59. 14633289 10.1186/1471-2105-4-59 PMC317282

[88] Meng C Kuster B Culhane AC A multivariate approach to the integration of multi-omics datasets. BMC Bioinformatics 2014;15:162. 24884486 10.1186/1471-2105-15-162 PMC4053266

[89] Tenenhaus A Philippe C Guillemot V Variable selection for generalized canonical correlation analysis. Biostatistics 2014;15:569–83. 24550197 10.1093/biostatistics/kxu001

[90] Jansen R Greenbaum D Gerstein M Relating whole-genome expression data with protein-protein interactions. Genome Res 2002;12:37–46. 11779829 10.1101/gr.205602 PMC155252

[91] Ideker T Ozier O Schwikowski B Discovering regulatory and signalling circuits in molecular interaction networks. Bioinformatics 2002;18 (Suppl 1):S233–40. 12169552 10.1093/bioinformatics/18.suppl_1.s233

[92] Guo Z Wang L Li Y Edge-based scoring and searching method for identifying condition-responsive protein-protein interaction sub-network. Bioinformatics 2007;23:2121–8. 17545181 10.1093/bioinformatics/btm294

[93] Louhimo R Hautaniemi S CNAmet: an R package for integrating copy number, methylation and expression data. Bioinformatics 2011;27:887–8. 21228048 10.1093/bioinformatics/btr019

[94] Tyekucheva S Marchionni L Karchin R Integrating diverse genomic data using gene sets. Genome Biol 2011;12:R105. 22018358 10.1186/gb-2011-12-10-r105 PMC3333775

[95] Shen R Wang S Mo Q Sparse integrative clustering of multiple omics data sets. Ann Appl Stat 2013;7:269–94. 24587839 10.1214/12-AOAS578 PMC3935438

[96] Huber W Carey VJ Gentleman R Orchestrating high-throughput genomic analysis with Bioconductor. Nat Methods 2015;12:115–21. 25633503 10.1038/nmeth.3252 PMC4509590

[97] Witten DM Tibshirani R Hastie T A penalized matrix decomposition, with applications to sparse principal components and canonical correlation analysis. Biostatistics 2009;10:515–34. 19377034 10.1093/biostatistics/kxp008 PMC2697346

[98] Liquet B Lê Cao K-A Hocini H A novel approach for biomarker selection and the integration of repeated measures experiments from two assays. BMC Bioinformatics 2012;13:325. 23216942 10.1186/1471-2105-13-325 PMC3627901

