# Supplementary material for: Public data and open source tools for multi-assay genomic investigation of disease
Source: Brief Bioinform. 2015 Oct 10;17(4):603–15. doi: 10.1093/bib/bbv080 (PMC4945830; doi:10.1093/bib/bbv080)
Supplement: Supplementary Data [file supp_17_4_603__index.html]

Public data and open source tools for multi-assay genomic investigation of disease — Public data and open source tools for multi-assay genomic investigation of disease — Supplementary Data 

# Public data and open source tools for multi-assay genomic investigation of disease

## Supplementary Data

files

- Supplementary Data - docx file
