# Supplementary material for: Public data and open source tools for multi-assay genomic investigation of disease
Source: Brief Bioinform. 2015 Oct 10;17(4):603–15. doi: 10.1093/bib/bbv080 (PMC4945830; doi:10.1093/bib/bbv080)
Supplement: Supplementary Data [file supp_bbv080_Supplementary_Table.docx]

# Supplemental Materials

| **Supplemental Table S1** | |
| --- | --- |
| Code | Disease |
| ACC | Adrenocortical carcinoma |
| BLCA | Bladder Urothelial Carcinoma |
| BRCA | Breast invasive carcinoma |
| CESC | Cervical squamous cell carcinoma and endocervical adenocarcinoma |
| CHOL | Cholangiocarcinoma |
| COAD | Colon adenocarcinoma |
| DLBC | Lymphoid Neoplasm Diffuse Large B-cell Lymphoma |
| ESCA | Esophageal carcinoma |
| GBM | Glioblastoma multiforme |
| HNSC | Head and Neck squamous cell carcinoma |
| KICH | Kidney Chromophobe |
| KIRC | Kidney renal clear cell carcinoma |
| KIRP | Kidney renal papillary cell carcinoma |
| LAML | Acute Myeloid Leukemia |
| LGG | Brain Lower Grade Glioma |
| LIHC | Liver hepatocellular carcinoma |
| LUAD | Lung adenocarcinoma |
| LUSC | Lung squamous cell carcinoma |
| MESO | Mesothelioma |
| OV | Ovarian serous cystadenocarcinoma |
| PAAD | Pancreatic adenocarcinoma |
| PCPG | Pheochromocytoma and Paraganglioma |
| PRAD | Prostate adenocarcinoma |
| READ | Rectum adenocarcinoma |
| SARC | Sarcoma |
| SKCM | Skin Cutaneous Melanoma |
| STAD | Stomach adenocarcinoma |
| TGCT | Testicular Germ Cell Tumors |
| THCA | Thyroid carcinoma |
| THYM | Thymoma |
| UCEC | Uterine Corpus Endometrial Carcinoma |
| UCS | Uterine Carcinosarcoma |
| UVM | Uveal Melanoma |

| **Supplemental Table S2** | |
| --- | --- |
| Name | Data Type |
| bisulfiteseq | Bisulfite Sequencing |
| clin | Clinical information |
| cna | Copy Number Alterations |
| exon | Exon |
| fragment_analysis | Microsatellite Instability (MSI) |
| methylation | Methylation |
| mirna | microRNA |
| mirnaseq | microRNA sequence |
| mutations | Mutations |
| mutations_protected | Mutations including whole genome |
| protein_exp | Protein expression |
| reports | Pathology reports |
| rnaseq | RNA sequence |
| rnaseqv2 | RNA sequencing algorithm version 2 |
| slide_images | Diagnostic/Tissue slide images |
| snp | Single nucleotide polymorphism |
| totalrnaseqv2 | mRNA sequence |
| tracerel | Trace-Sample Relationship |
| transcriptome | RNA Transcriptome |
